# 11. Electronic Surveillance Criteria for Non-Ventilator HAP: Empiric testing and Chart Review at Veterans Affairs Facilities

**DOI:** 10.1093/ofid/ofab466.011

**Published:** 2021-12-04

**Authors:** Sarah Stern, Matthew A Christensen, McKenna Nevers, Jian Ying, Caroline Smith, Robert Jin, Aileen Ochoa, Chanu Rhee, Matthew H Samore, Michael Klompas, Barbara E Jones

**Affiliations:** 1 Division of Pulmonary & Critical Care Medicine, Department of Internal Medicine, University of Utah School of Medicine, Salt Lake City, Utah; 2 University of Utah Health, Salt Lake City, UT; 3 Division of Epidemiology, Department of Internal Medicine, Harvard Pilgrim Institute, Boston, Massachusetts; 4 Harvard Medical School and Harvard Pilgrim Health Care Institute, Boston, Massachusetts; 5 Harvard Medical School and Harvard Pilgrim Health Institute, Boston, MA; 6 VA Salt Lake City Healthcare System, Salt Lake City, Utah

## Abstract

**Background:**

Surveillance of Non-Ventilator Hospital-Acquired Pneumonia (NV-HAP) is limited by the ambiguity in diagnosing pneumonia. We implemented electronic surveillance criteria for NV-HAP across the VA healthcare system and tested for reliability, validity and meaning of the electronic criteria vs manual chart review.

**Methods:**

We defined NV-HAP surveillance criteria as oxygen deterioration concurrent with fever or abnormal WBC count, ≥3 days of antibiotics, and orders for chest imaging. We applied these criteria to EHR data from all patients hospitalized ≥3 days at all VA acute care facilities from 1/1/2015-12/31/2020 and calculated NV-HAP incidence and inpatient mortality. Clinician reviewers used a consensus review guide to independently review and adjudicate 47 cases meeting NV-HAP surveillance criteria for 1) clinical deterioration, 2) CDC-NHSN pneumonia criteria, 3) treating clinicians’ assessment, and 4) reviewer’s diagnosis. All reviewers subsequently adjudicated all cases and conducted an error analysis to identify sources of discordance.

**Results:**

Among 2.3M hospitalizations, 14,023 met NV-HAP surveillance criteria (0.6 per 100 admissions). Inpatient mortality was 26% (vs 2% for non-flagged hospitalizations). Among 47 hospitalizations flagged by surveillance criteria, 45 (97%) had a confirmed clinical deterioration, (the other 2 were immediate post-operative cases), 20 (43%) met CDC-NHSN pneumonia criteria, 21 (47%) had possible pneumonia per treating clinicians, and 25 (53%) had possible or probable NV-HAP per reviewers. Agreement among the 3 reviewers before adjudication was 51% (Fleiss’ κ 0.43) for CDC-NHSN and 58% (Fleiss’ κ 0.33) for NV-HAP. The most common source of discordance between reviewers was chest imaging classification (15/19 discordant cases).

**Conclusion:**

NV-HAP electronic surveillance criteria demonstrated high precision for identifying clinical deterioration and moderate concordance with CDC-NHSN pneumonia criteria or reviewer diagnosis. Agreement between electronic surveillance criteria vs manual chart review was low but similar to agreement amongst manual reviewers applying NHSN criteria. Electronic surveillance may provide greater consistency than human review while facilitating wide-scale automated surveillance.

**Disclosures:**

**Chanu Rhee, MD, MPH**, **UpToDate** (Other Financial or Material Support, Chapter Author) **Michael Klompas, MD, MPH**, **UpToDate** (Other Financial or Material Support, Chapter Author)

